# The Means by Which the Temperature of the Body Is Maintained in Health and Disease

**Published:** 1897-06-19

**Authors:** W. Hale White

**Affiliations:** Physician to, and Lecturer on Pharmacology at, Guy's Hospital


### MEDICAL PROGRESS AND HOSPITAL CLINICS.

[*The Editor will be glad to receive offers of co-operation and contributions from members of the profession. All letters should be addressed to* THE EDITOR, AT THE OFFICE, 28 & 29, SOUTHAMPTON STREET, STRAND, LONDON, W.C.]

### THE MEANS BY WHICH THE TEMPERATURE OF THE BODY IS MAINTAINED IN HEALTH AND DISEASE.

THE CROONIAN LECTURES, delivered before the Royal College of Physicians of London.

By W. HALE WHITE, M.D., F.R.C.P. Lond., Physician to, and Lecturer on Pharmacology at, Guy's Hospital.

LECTURE I.—Delivered on June 15th.

After expressing his thanks for the honour conferred upon him in being invited to deliver the Croonian Lectures, Dr. Hale White said that he proposed to discuss in some detail the means by which the temperature of the body was maintained in health and disease. It would be noted that he had avoided the use of the word "fever" in the title of these lectures. This as commonly used was taken by most people to mean a certain assemblage of symptoms which follow the introduction into the body of certain poisons; thus when an unprotected person was inoculated with small-pox he showed certain symptoms, and was therefore said to have fever. One of the most prominent of these symptoms was pyrexia, but pyrexia might occur, as he would show, if we remained too long in a hot bath, or as a result of cerebral hæmorrhage; and, although under such conditions we could not be said to have fever, before we could understand the pyrexia which occurred in course of febrile diseases, it was necessary that we should know the manner in which pyrexia was produced in these less complex conditions.

In studying pyrexia experimentally it was necessary to beware of certain pitfalls.

In the first place, the normal range of temperature was wider in the lower animals than in man. Then the prolonged administration of an anæsthetic caused a fall of temperature. Another fallacy arose from the fact that the performance of artificial respiration of itself caused a considerable alteration in the heat of the body. Even tying an animal down, by which its respiration might be interfered with, might cause the temperature to fall. From experiments which he had made along with Dr. Fawcett, he had found that combined etherisation and artificial respiration produced a lowering of temperature which could not be prevented by wrapping the animals in cotton wool.

The fundamental problem in the study of pyrexia was to discover whether or not the production of heat was increased, for it was clear that the temperature of the body might rise either because the loss of heat from it was less, while the production remained the same, or because the loss of heat remained the same while its production was increased; or, if both varied, because the variations in production had not been completely counter-balanced by variation in loss.

Great differences of opinion had existed in regard to this question, but it seemed to him that these arose to some extent from the fact that observers had studied "fever," rather than pyrexia pure and simple. Much of the discussion that had taken place on this subject among authorities had in a sense been beside the mark, for we knew that most forms were due to the circulation of poisons in the blood, and there was no reason for believing that all these poisons produced pyrexia in the same way. All collateral evidence was against such a view. Many poisons, for example, accelerated the action of the heart, but some did this by acting on the vagus, others on the accelerator nerves; many poisons produced diaphoresis, but not always in the same way; and he hoped to be able to show that sometimes pyrexia was due to an increase in the production of heat and sometimes to a diminution of the loss. If that could be done it would no longer be to the point to discuss whether in fever at large heat production was increased or heat-loss was diminished. Each febrile disease would have to be considered separately and by itself.

An interesting preliminary question, however, arose in regard to the specific heat of the body. The problem might be put in this way: It takes more heat units to warm to a given temperature a body with a high specific heat than it does to warm a body with a low specific heat to the same temperature, so that if the specific heat of the human body diminished while its production and loss of heat remained the same its temperature would rise; and at first sight it appears that this would be a most economical way of raising the temperature, as there would be no extra expenditure of energy in increasing the production of heat; but perhaps the alteration of the specific heat of the body might entail such considerable molecular change that it would necessitate an expenditure of energy to produce it.

Many carefully-conducted experiments were described which had been undertaken to decide this point, and the conclusion was arrived at (*a*) that the specific heat of the body was high; (*b*) that it was an advantage to warm-blooded animals that this should be the case, as they were thereby less rapidly affected by variations of surrounding temperature than otherwise would be the case; (*c*) that there was no evidence that in man the rise of temperature in pyrexia was due to a diminution of the specific heat, and (*d*) that the specific heat of the body increased with the temperature, a fact which tended to facilitate heat regulations, since, other things being equal, it would take a few more calories of heat, and therefore require a greater production of heat to raise the temperature from 104 deg. to 105 deg. than it would to raise it from 98 deg. to 99 deg.

To solve the problem whether or not in pyrexia the production of heat was increased or the loss diminished, Dr. Hale White then described the proceedings which he had adopted to compare in an accurate manner the variations of the loss of heat with variations of internal temperature. It was clear, he said, that when the surface temperature of the body rose the loss of heat by radiation and evaporation would be increased, as also it would when the secretion of perspiration was increased, so that if in any case it could be shown that the internal temperature had risen, and that at the same time the surface temperature had risen, and the amount of sweat secreted had increased, the production of heat must also have increased, provided the temperature of the surrounding air was constant, which in the wards of a hospital was approximately the case. The results of his observations he would give in the next lecture.

